# One-Stool Veterinary Colleges

**Published:** 1883-04

**Authors:** 


One-stool Veterinary Colleges.—We continue to insist upon the inutility of establishing so-called veterinary departments with one or two professors in connection with a large university or medical school. A thorough veterinary education cannot be given in this way. We learn that an epidemic of this sort of thing is threatened. It is thought a cheap way starting a veterinary college.

